# Differences in nulliparous caesarean section rates across models of care: a decomposition analysis

**DOI:** 10.1186/s12913-016-1494-3

**Published:** 2016-07-08

**Authors:** Aoife Brick, Richard Layte, Anne Nolan, Michael J. Turner

**Affiliations:** Economic and Social Research Institute, Whitaker Square, Sir John Rogerson`s Quay, Dublin 2, Ireland; Trinity College, Dublin, Ireland; Department of Sociology, Trinity College, Dublin 2, Ireland; UCD Centre for Human Reproduction, Coombe Women and Infants University Hospital, Dublin 8, Ireland

**Keywords:** Elective caesarean section, Emergency caesarean section, Private practice, Non-linear Oaxaca–Blinder decomposition method

## Abstract

**Background:**

To evaluate the extent of the difference in elective (ELCS) and emergency (EMCS) caesarean section (CS) rates between nulliparous women in public maternity hospitals in Ireland by model of care, and to quantify the contribution of maternal, clinical, and hospital characteristics in explaining the difference in the rates.

**Methods:**

Cross-sectional analysis using a combination of two routinely collected administrative databases was performed. A non-linear extension of the Oaxaca-Blinder method is used to decompose the difference between public and private ELCS and EMCS rates into the proportion explained by the differences in observable maternal, clinical, and hospital characteristics and the proportion that remains unexplained.

**Results:**

Of the 29,870 babies delivered to nulliparous women, 7,792 were delivered via CS (26.1 %), 79.6 % of which were coded as EMCS. Higher prevalence of ELCS was associated with breech presentation, other malpresentation, and the mother being over 40 years old. Higher prevalence of EMCS was associated with placenta praevia or placental abruption, diabetes (pre-existing and gestational), and being over 40 years old. The private model of care is associated with ELCS and EMCS rates 6 percentage points higher compared than the public model of care but this differential is insignificant in the fully adjusted models for EMCS. Just over half (53 %) of the 6 percentage point difference in ELCS rates between the two models of care can be accounted for by maternal, clinical and hospital characteristics. Almost 80 % of the difference for EMCS can be accounted for.

**Conclusions:**

The majority of the difference in EMCS rates across models of care can be explained by differing characteristics between the two groups of women. The main contributor to the difference was advancing maternal age. The unexplained component of the difference for ELCS is larger; an excess private effect remains after accounting for maternal, clinical, and hospital characteristics. This requires further investigation and may be mitigated in future with the introduction of clinical guidelines related to CS.

**Electronic supplementary material:**

The online version of this article (doi:10.1186/s12913-016-1494-3) contains supplementary material, which is available to authorized users.

## Background

In recent decades a feature of modern obstetric practice in developed countries has been the relentless rise in caesarean section (CS) rates. The reasons for the increase in CS rates across countries are multiple and complex but have been attributed to the increasing prevalence of older mothers, rising rates of maternal obesity, medical comorbidities, and changing medical practice including the increasing relative safety of CS itself [1–4].

While CS rates are rising globally, there is substantial evidence that this increase is more prevalent amongst women with privately funded deliveries [5–11]. This raises questions of whether CS is always medically indicated in privately funded patients [7, 8]. There is an important issue of confounding in this scenario however. Women with a higher risk of complicated pregnancies and deliveries due to older maternal age and other relevant maternal and clinical risk factors are more likely to take out private health insurance (PHI) due to perceived quality of care [12–17]. The positive association between private care and CS may thus reflect the selection of women with clinically complex deliveries into private care rather than measuring the effect of private status on the process leading to CS.

As well as women selecting private status there may also be clinician/ organisational level factors at play. For example, with high numbers of deliveries to contend with the ability to be able to schedule deliveries for private patients may become increasingly attractive [18]. In addition, in an increasingly litigious society the level of risk a clinician deems acceptable may be lower, particularly so for private patients [19].

Models of obstetric care in Ireland are unusual by international standards in that private patients are looked after by the same midwifery staff in the wards, including the labour ward, as public patients. Furthermore, the same clinical guidelines are applied across models of care. Nonetheless, variation in obstetric interventions across models of care has been found. The rate of increase in CS for women funded publicly was 7.2 % (22.2 % to 23.8 %) between 2005 and 2010 in Ireland compared to 14.9 % (30.2 % to 34.7 %) for those funded privately [19]. Irish research suggests that the higher rate of CS for privately funded births cannot be explained by differences in medical or obstetric risk factors between women but no study to date has employed national, individual data to test this hypothesis [20, 21].

Research internationally has suggested that financial incentives may increase the probability of CS [9, 22, 23] but the payment method for private care in Irish hospitals and hospital financing system means that the financial benefit from higher CS rates does not accrue to the hospital. In fact, the annual financial allocation to hospitals is reduced as income from private practice increases.

Previous literature has described the increasing prevalence of CS and the role of the private model of care in this. This paper contributes to the literature by quantifying the extent to which the differences in CS rates across models of care is due to differences in maternal, clinical, and hospital characteristics. To reduce the heterogeneity of the sample and the complexity of the processes that we study, this paper focuses solely on nulliparous women.

Section 2 describes the data, Section 3 outlines the methods, Section 4 presents empirical results, and Section 5 discusses the findings and concludes.

## Methods

### Data

The data for these analyses were obtained from two national databases in the Republic of Ireland, the National Perinatal Reporting System (NPRS) and the Hospital In-Patient Enquiry (HIPE) scheme. The main source of data on perinatal events in Ireland is the NPRS which contains information on all deliveries (≥500 g) in the Republic of Ireland. Unfortunately, clinical data on deliveries are not available in the NPRS which means that it is not possible to identify the clinical indicators for CS using these data, including whether the CS was carried out on an elective or emergency basis.

However, data on these variables are available from the HIPE system. The HIPE system records data on all discharges from, and deaths in, acute publicly funded hospitals in the Republic of Ireland. These data contain information on administrative data such as admission and discharge dates and model of care (public/private) as well as maternal and child demographic data and clinical information. The clinical data on discharges in 2009 were recorded in HIPE using the 6th Edition of *The International Statistical Classification of Diseases and Related Health Problems*, Tenth Revision, Australian Modification (ICD-10-AM), the *Australian Classification of Health Interventions* (ACHI) and the Australian Coding Standards (ACS). Deliveries in HIPE are identified by the presence of a diagnosis of *outcome of delivery* (ICD-10-AM – Z37). In total, up to 20 diagnosis codes (one principal and up to 19 additional) and, where applicable, 20 procedure codes (one principal and up to 19 additional) could be recorded for these discharges.

The sample employed for these analyses consists of all nulliparous singleton births to women discharged from the 19 publicly funded hospital maternity units between 01 January 2009 and 31 December 2009 and for whom an NPRS and HIPE record were available; a total of 29,870 births. This represented 96.7 % of the total number of nulliparous singleton births in publicly funded hospital maternity units in 2009. For these analyses, only births (live and stillborn) that took place within a hospital were included, that is, cases where ‘place of birth’ is recorded as ‘domiciliary’, ‘born before arrival’ and ‘unknown’ are excluded.

### Dependent variables

The dependent variables are binary indicators of whether the mother had a CS (elective or emergency) or not. In ACS (1541) an ELCS (ACHI 16520–00, 16520–02) is defined as a CS carried out as a planned procedure before the onset of labour or following the onset of labour, when the decision was made before labour. An EMCS (ACHI 16520–01, 16520–03) is defined as a CS required because of an emergency situation. It is best described as ‘when the CS is performed having not been considered necessary previously’ [24]. According to the European Perinatal Health Report in 2010 these definitions are commonly used in other countries [25].

### Independent variables

Independent variables include those familiar from previous research on CS determinants [3]. Data on the woman‘s marital status, socio-economic group, country of birth, birthweight, gestational age, and obstetric history were sourced from NPRS. Data on the woman’s age, public/private status, method of delivery, and clinical risk factors for CS were sourced from HIPE. Socio-economic group is derived from information on maternal occupation, and coded, with minor modifications, using the schema employed by the Central Statistics Office. The clinical risk factors for CS included in the analysis are those commonly used throughout the literature (see Additional file 1 for codes). Fetal distress and dystocia have been excluded as indicators for EMCS. A variable representing whether the woman has previously experienced a miscarriage is included to measure the possible selection of women into private care as well as the direct correlation between past miscarriage and CS currently.

The main independent variable of interest is model of care which refers in these data to whether the woman was a public or private patient of their chosen consultant and not to the type of bed occupied. All women in Ireland are entitled to free maternity services but a proportion choose to finance their care privately through PHI and/or an ‘out-of-pocket’ payment. A public patient typically receives shared care from their family doctor and their chosen hospital. They may or may not see the same obstetrician on each of their antenatal visits to the hospital and after delivery is moved to a shared room (usually four or six bedded).

Being a private patient in a publicly funded hospital means that you are the private patient of your chosen consultant who will supervise your antenatal care. The consultant or a nominated consultant colleague will also attend for delivery. After delivery, patients are transferred to a private room if one is available. Some hospitals also offer a semi-private option, which usually means patients antenatal care alternates between their chosen consultant and their GP. Their delivery will be attended by the obstetrician on duty and they are transferred to a semi-private room following delivery if one is available. In HIPE, unfortunately both private and semi-private patients are classified as private and in the 2009 data it is not possible to distinguish between the two. This could mean that the effect of private care is reduced if the processes associated with private care among women do not apply to semi-private care.

### Statistical analysis

#### Determinants of CS

We first analyse the determinants of ELCS and EMCS by estimating the following non-linear model:

1 $$ {Y}_i=F\left({X}_i\beta \right) $$

where *y*_*i*_ is the dummy variable which indicates whether the woman had a CS and *X*_*i*_ is a vector of maternal and clinical characteristics.

### Decomposition of the difference in cs rates across models of care

To examine the extent to which the differences in the rates of ELCS and EMCS across models of care is due to differences in their observed characteristics, a non-linear approximation of the Blinder-Oaxaca decomposition technique is used [26–28]. The technique is used to study group differences in an outcome variable and has been employed in analyses of racial/ethnic differences in birthweight; child mortality; child health insurance cover; life expectancy; and breastfeeding [29–34].

The average difference in the CS rates between public and private women may be expressed as:

2 $$ {\overline{Y}}^{pub}-{\overline{Y}}^{pri}=\left[{\displaystyle {\sum}_{i=1}^{N^{pub}}\frac{F\left({X}_i^{pub}{\widehat{\beta}}^{pub}\right)}{N^{pub}}-}{\displaystyle {\sum}_{i=1}^{N^{pri}}\frac{F\left({X}_i^{pri}{\widehat{\beta}}^{pub}\right)}{N^{pri}}}\right]+\left[{\displaystyle {\sum}_{i=1}^{N^{pri}}\frac{F\left({X}_i^{pri}{\widehat{\beta}}^{pub}\right)}{N^{pri}}-}{\displaystyle {\sum}_{i=1}^{N^{pri}}\frac{F\left({X}_i^{pri}{\widehat{\beta}}^{pri}\right)}{N^{pri}}}\right] $$

where ${\overline{Y}}^J$ is the average probability of CS for group *J* (*J = pub, pri*) *X*_*i*_^*J*^ is the vector of independent variables of observation *i* in group *J*, ${\widehat{\beta}}^J$ is the vector of coefficient estimates and *N*^*J*^ is the number of observations in group *J*. In this case, group *pub* is the sample of public mothers, group *pri* is the sample of private mothers, and the reference is group *pub*. We also undertake the decomposition using the estimated coefficients of group *pri* and the pooled coefficients as the reference [29, 31, 35, 36].

In this application, the first term on the right hand side of (2) measures the amount of the gap in the CS rate that is due to differences in the characteristics of the two groups. The second term captures the degree to which public and private mothers with similar observable characteristics have different CS rates. This may be interpreted as reflecting, varying obstetric practice, group-specific attitudes, or other omitted variables. The first part may be further decomposed into the relative contributions of each of the observed independent variables. We use the ‘Fairlie’ decomposition command in STATA 13.1 [37] with randomly ordered variables and 1,000 replications [38].

## Results

Table 1 shows prevalence and unadjusted CS rates by model of care by maternal, clinical, and hospital characteristics. There were higher proportions of private patients in the older age groups. Over 80 % of private patients were in the professional/managerial or clerical social classes (*p* < 0.01). Only 45 % of public women were married compared to 83.5 % of private women (*p* < 0.01). Over 92 % of private women were born in Ireland compared to 64 % of public women. Of the public women born outside Ireland, 20.9 % were from the EU-27 countries (*p* < 0.01).

**Table 1:** Prevalence and Unadjusted Caesarean Section Rates (Elective and Emergency) for Nulliparous Singleton Deliveries by Maternal and Clinical Characteristics, 2009

	Prevalence			CS Rate^a^			
				Elective		Emergency	
	Public	Private	*p*-value	Public	Private	Public	Private
N	22,059	7,811		913	664	4,325	1,890
CS Rate	23.7	32.7		4.1	8.5	19.6	24.2
	%	%		%	%	%	%
Maternal Characteristics							
Age (years)							
<20	8.9	0.5	0.000***	2.1	0.0	12.0	7.7
20–24	25.0	1.8		2.6	4.4	16.2	15.3
25–29	34.2	17.8		3.8	5.5	18.5	21.6
30–34	22.9	52.5		5.1	7.5	23.5	23.1
35–39	7.9	23.3		7.6	10.9	30.7	28.4
≥40	1.2	4.1		19.6	23.2	31.7	31.0
Social Class							
Professional/managerial	21.2	56.5	0.000***	4.2	8.5	21.7	24.1
Clerical	26.0	27.9		4.9	9.0	20.3	25.1
Skilled/semi-skilled	7.3	3.5		4.9	9.9	19.1	22.6
Unskilled	18.2	7.3		4.1	7.9	19.8	24.3
Unemployed	4.6	0.2		3.6	5.9	16.1	23.5
Home duties	15.1	1.9		3.4	6.9	19.0	25.5
Other^b^	7.5	2.8		2.5	5.1	14.9	18.9
Marital Status							
Married	44.9	83.5	0.000***	4.9	8.8	21.2	24.2
Not married^c^	55.1	16.5		3.5	7.2	18.4	24.2
Country of Birth							
Ireland	64.2	92.1	0.000***	4.3	8.4	20.7	24.2
UK	2.4	1.6		5.4	14.2	18.5	19.7
EU-15	1.8	2.1		5.7	9.9	19.5	25.3
EU-27^d^	20.9	1.2		3.8	6.4	14.9	18.1
Africa	2.4	0.2		3.2	12.5	27.9	31.3
Asia	5.9	1.1		3.2	11.4	21.9	21.6
Other^e^	2.4	1.7		4.1	8.5	17.7	31.8
Clinical Characteristics							
Obstetric History							
Previous miscarriage	13.3	17.9	0.000***	5.8	9.7	23.1	26.8
Gestational Age (weeks)							
<33	1.4	1.2	0.460	4.6	3.2	38.5	54.8
33–37	7.8	7.9		5.2	11.4	25.8	27.8
38+	90.8	90.9		4.0	8.3	18.8	23.5
Birthweight (g)							
500–2499	5.0	4.1	0.000***	6.4	8.5	35.3	45.1
2500–2999	13.9	11.2		4.5	10.1	17.3	23.7
3000–3499	36.5	34.5		4.6	8.8	15.2	19.6
3500–3999	32.4	35.3		3.2	7.8	18.9	22.4
4000–4499	10.5	12.5		3.1	7.2	29.1	31.4
4500+	1.7	2.4		6.9	13.6	40.8	46.7
Clinical Risk Factors^f^							
Breech presentation	4.1	4.9	0.005***	66.0	72.6	29.2	25.3
Diabetes mellitus (pre-existing)	0.3	0.2	0.025**	10.7	35.7	50.7	50.0
Gestational diabetes mellitus	1.7	1.0	0.000***	6.8	23.8	33.7	26.3
Eclampsia including pre-eclampsia	3.2	3.5	0.217	5.2	9.3	43.5	52.6
Other malpresentation	0.7	2.0	0.000***	35.4	70.9	43.5	23.4
Placenta praevia or placental abruption	0.6	0.9	0.051*	20.4	47.8	59.2	47.8
Hypertensive disorders	4.7	5.0	0.450	3.3	10.1	31.3	32.6
Poor fetal growth	2.5	2.3	0.353	10.4	15.6	32.0	33.3
Induction of labour	28.9	34.4	0.000***			27.3	32.9
Hospital Characteristics							
Academic teaching hospital	47.5	60.9	0.000***	4.0	8.5	18.8	21.6

Percentages columns subject to rounding. Missing values are excluded from the calculation of percentages. ***p < 0.01, **p < 0.05, *p < 0.1

^a^CS rates are calculated as a proportion of total deliveries

^b^Includes farmers and farm managers, other agricultural occupations and fisheries workers, and not classifiable

^c^Includes never married, divorced, separated, and widowed

^d^Accession States

^e^Includes the Rest of Europe, the Americas, Australia, New Zealand (incl. Oceania), multi-nationality, non-Irish, and no nationality

^f^Clinical risk factors imply that the relevant diagnosis code(s) appeared as one of the up to 20 diagnosis codes (one principal and up to 19 additional) recorded in HIPE, a woman may have more than one clinical risk factor. The presence of a particular risk factor does not imply that this was the ‘cause’ of the CS

Source: HIPE/NPRS 2009 data

Almost 18 % of private women had a previous miscarriage compared to 13.3 % of public women (*p* < 0.01). Women in the public group had smaller babies than those in the private group (*p* < 0.01). Many of the clinical characteristics varied significantly by model of care including breech presentation, diabetes mellitus (pre-existing and gestational), other malpresentation, placenta praevia or placental abruption and induction of labour. A majority of private women (60.9 %) gave birth in an academic teaching hospital compared to public women (47.5 %) (*p* < 0.01).

A key objective of this research is to examine the extent to which the difference in CS rates across models of care can be accounted for by the differences in maternal, clinical, and hospital characteristics. That is, do private women have higher CS rates primarily because they have socio-demographic and clinical characteristics that are associated with higher CS rates?

**Fig. 1:**
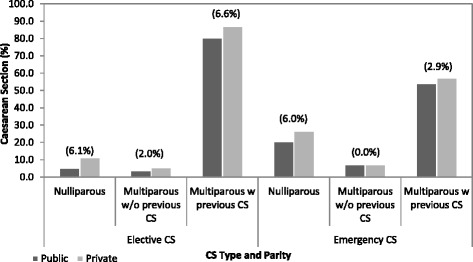
Elective and Emergency Caesarean Section by Model of Care and Parity, 2009. Notes: Percentage point differences between the public and private rates in parentheses. The ELCS rate is calculated here excluding EMCS and vice versa. Source: HIPE/NPRS 2009 data

Figure 1 shows that there is a 6 percentage point difference between both the ELCS and EMCS rates for public and private women. The difference between rates varied across model of care with the presence of various characteristics. For ELCS the private rate is higher than the public rate for almost all characteristics. The differences are particularly large for the clinical risk factors. For EMCS there is more variation with the public rate being higher than the private rate for some indications. For example, the public ELCS rate for women with other malpresentation is 35.4 % compared to 70.9 % for private women. In contrast, for the same indication, the EMCS rate is 43.5 % for public women and 23.4 % for private women.

### Determinants of CS

The results from the probit models for ELCS and EMCS follow a similar pattern to the descriptive statistics discussed earlier. Table 2 shows that in the fully adjusted models, women who are privately funded have a significantly higher probability of ELCS compared to publicly funded women, but also that private status has no significant effect on EMCS risk. The probability of both EMCS and ELCS increases significantly with maternal age and where mother is defined as holding a clerical or unskilled occupation or is a full-time carer relative to women with a professional occupation. In addition, being unemployed increases the probability of ELCS but has no effect on EMCS.

**Table 2:** Determinants of Caesarean Section (Elective and Emergency) at Hospital Discharge, 2009

	Elective				Emergency			
	(1)		(2)		(1)		(2)	
Public (REF)								
Private	0.053		0.019		0.057		0.011	
	(0.016)	***	(0.005)	***	(0.016)	***	(0.013)	
Age								
Mother Age (years)			0.003				0.009	
			(0.001)	***			(0.001)	***
Social Class								
Professional/managerial (REF)								
Clerical			0.007				0.013	
			(0.002)	***			(0.004)	***
Skilled/semi-skilled			0.008				0.006	
			(0.006)				(0.011)	
Unskilled			0.007				0.017	
			(0.002)	***			(0.009)	*
Unemployed			0.013				0.016	
			(0.007)	*			(0.017)	
Home duties			0.015				0.025	
			(0.003)	***			(0.010)	***
Other^a^			0.010				−0.012	
			(0.007)				(0.008)	
Marital Status								
Married (REF)								
Not married^b^			0.001				0.014	
			(0.003)				(0.006)	**
Country of Birth								
Ireland (REF)								
UK			0.012				−0.020	
			(0.007)	*			(0.014)	
EU-15 (excl. IRE & UK)			−0.002				−0.013	
			(0.005)				(0.018)	
EU-27 Accession States^c^			0.002				−0.043	
			(0.004)				(0.007)	***
Africa			0.017	*			0.094	
			(0.009)				(0.009)	***
Asia			−0.012				0.024	
			(0.006)	**			(0.010)	**
Other^d^			−0.002				0.001	
			(0.006)				(0.013)	
Obstetric History								
Previous miscarriage			0.004				0.018	
			(0.003)				(0.007)	***
Baby Characteristics								
Birthweight (kg)			0.006				0.043	
			(0.002)	***			(0.010)	***
Clinical Risk Factors^e^								
Breech			0.197				0.586	
			(0.022)	***			(0.029)	***
Diabetes (pre-existing)			0.062				0.260	
			(0.014)	***			(0.045)	***
Eclampsia or pre-eclampsia			0.032				0.203	
			(0.009)	***			(0.015)	***
Gestational diabetes			0.032				0.092	
			(0.007)	***			(0.024)	***
Hypertensive disorders			0.005				0.087	
			(0.004)				(0.013)	***
Malpresentation			0.143				0.354	
			(0.020)	***			(0.050)	***
Placenta praevia or placental abruption			0.120				0.438	
			(0.012)	***			(0.028)	***
Poor fetal growth			0.046				0.111	
			(0.009)	***			(0.027)	***
Induction							0.081	
							(0.017)	***
Hospital Characteristics								
Academic teaching hospital			−0.006				−0.047	
			(0.006)				(0.015)	***
N	23,655		23,515		28,293		28,136	
Psuedo R^2^	0.020		0.594		0.004		0.097	

Results are presented as marginal effects, with robust standard errors in parentheses. Column (1) for each includes controls for private only, while column (2) adds all other variables. *** *p* < 0.01, ** *p* < 0.05, * *p* < 0.1

^a^ Includes farmers and farm managers, other agricultural occupations and fisheries workers, and not classifiable

^b^ Includes never married, divorced, separated, and widowed

^c^ Accession States

^d^ Includes the Rest of Europe, the Americas, Australia, New Zealand (incl. Oceania), multi-nationality, non-Irish, and no nationality

^e^ Clinical risk factors imply that the relevant diagnosis code(s) appeared as one of the up to 20 diagnosis codes (one principal and up to 19 additional) recorded in HIPE, a woman may have more than one clinical risk factor. The presence of a particular risk factor does not imply that this was the ‘cause’ of the CS

Source: HIPE/NPRS 2009 data

Not being married is associated with an increased probability of EMCS compared to married woman. Women born in Africa have a significantly higher risk of both EMCS and ELCS whilst women from Asia are at a higher risk of EMCS but a significantly lower risk of ELCS relative to women from Ireland. Being from EU-27 Accession States significantly lowers the probability of EMCS. Women born in the UK have a significantly higher probability of having an ELCS compared to Irish-born women.

Having had a previous miscarriage had a significant positive impact on the probability of EMCS. Birthweight and all included clinical risk factors have significantly positive effects on the probability of both ELCS and EMCS with the exception of hypertensive disorders on ELCS which has no effect. Delivering in an academic teaching hospital significantly reduces the probability of having an EMCS but has no significant effect on ELCS.

### Decomposition of the difference in CS rates across models of care

To investigate if the socio-demographic and clinical risk profile of private women accounts for their higher risk of CS, a decomposition of the difference in ELCS and EMCS rates between public and private women was undertaken, the results of which are presented in Table 3. The raw difference in CS rates is 6.1 percentage points for ELCS and 6.0 percentage points for EMCS, with private women having the higher rates. The results show that the distribution of age, breech presentation and malpresentation between private and public women accounts for 53.3 % of the difference in ELCS rate and 79.7 % of the difference in the EMCS rate. Examining the results using different reference coefficients reveals that while the size of the explained contribution differs (ELCS private 71.1 %, pooled 54.9 %; EMCS private 87.9 %, pooled 78.2 %), the differences are not large, and the main conclusions still hold [28, 31, 35].

**Table 3:** Decomposition of the Differential in Elective and Emergency Caesarean Section Rates between Public and Private Mothers

	Elective				Emergency^a^			
	% pt diff	% of ${\overset{-}{Y}}^{pub}-{\overset{-}{Y}}^{pri}$	z-stat		% pt diff	% of ${\overset{-}{Y}}^{pub}-{\overset{-}{Y}}^{pri}$	z-stat	
${\overset{-}{Y}}^{pub}-{\overset{-}{Y}}^{pri}$	−0.061				−0.060			
Explained^b^	−0.032	53.3			−0.047	79.7		
Unexplained^c^	−0.028	46.7			−0.012	20.3		
Mother Age	−0.012	18.9	−7.8	***	−0.048	80.6	−14.9	***
Social Class								
Professional/managerial (REF)								
Clerical	0.000	0.3	−1.1		0.000	0.4	−1.3	
Skilled/semi-skilled	0.000	−0.7	1.5		0.000	−0.7	0.9	
Unskilled	0.001	−1.4	1.7	*	0.002	−4.0	2.3	**
Unemployed	0.000	−0.8	1.4		0.001	−1.0	0.9	
Home duties	0.002	−3.4	2.7	***	0.004	−6.5	2.9	***
Other^d^	0.000	−0.8	1.6	*	0.000	0.5	−0.6	
Marital Status								
Married (REF)								
Not married^e^	−0.001	1.0	−0.6		0.005	−7.7	1.9	*
Country of Birth								
Ireland (REF)								
UK	0.000	−0.1	0.6		0.000	0.3	−1.2	
EU-15 (excl. IRE & UK)	0.000	0.0	0.0		0.000	−0.1	0.5	
EU-27 Accession States^f^	0.000	0.7	−0.7		−0.008	13.7	−6.3	***
Africa	0.000	−0.8	1.5		0.002	−4.1	4.9	***
Asia	0.000	0.6	−2.0	**	0.001	−2.2	1.9	*
Other^g^	0.000	0.2	−1.5		0.000	0.4	−1.6	
Obstetric History								
Previous miscarriage	0.000	0.4	−1.7	*	−0.001	1.3	−2.0	**
Baby Characteristics								
Birthweight	−0.001	1.5	−2.9	***	−0.004	6.4	−8.7	***
Clinical Risk Factors^h^								
Breech	−0.009	14.8	−36.6	***	0.000	−0.2	1.6	
Diabetes (pre-existing)	0.000	−0.1	1.0		0.001	−0.9	5.5	***
Eclampsia or pre-eclampsia	0.000	−0.6	1.9	*	−0.001	1.6	−8.8	***
Gestational diabetes	0.001	−0.8	3.0	***	0.001	−1.8	4.9	***
Hypertensive disorders	0.000	0.0	−0.1		0.000	0.5	−3.7	***
Malpresentation	−0.013	21.5	−19.1	***	−0.001	1.0	−5.0	***
Placenta praevia or placental abruption	−0.002	3.7	−9.0	***	0.000	−0.2	1.2	
Poor fetal growth	0.000	0.0	0.1		0.000	−0.2	1.5	
Induction					−0.007	12.0	−12.0	***
Hospital Characteristics								
Academic teaching hospital	0.000	−0.8	1.1		0.006	−9.6	6.8	***

Results are presented as marginal effects, with robust standard errors in parentheses

Using the public (pub) coefficients as the reference. Using the private (pri) coefficients as the reference, the explained components were 84.4 % for elective and 82.2 % for emergency, while using the pooled coefficients results in an explained component of 58.6 % for elective and 78.3 % for emergency (full results available on request from the authors)

*** *p* < 0.01, ** *p* < 0.05, * *p* < 0.1

^a^ Robustness checks were carried out on the EMCS model by excluding clinical characteristics and the proportion explained by the model increased (87.3 % explained). In particular the proportion explained by age and EU-27. The EMCS model was also run excluding age and the proportion explained by the model halves (44.7 % explained). This highlights that age explains a large proportion of the difference between public and private EMCS rates and when removed from the model it becomes much less powerful. This is not to say that age is not picking up some omitted variables such as BMI. Results available on request

^b^ The differential that is estimated based on differences in observed characteristics (i.e., the first term in Equation (2))

^c^ The differential that is estimated based on differences in omitted variables e.g. BMI, smoking, etc. (i.e., the second term in Equation (2))

^d^ Includes farmers and farm managers, other agricultural occupations and fisheries workers, and not classifiable

^e^ Includes never married, divorced, separated, and widowed

^f^ Accession States

^g^ Includes the Rest of Europe, the Americas, Australia, New Zealand (incl. Oceania), multi-nationality, non-Irish, and no nationality

^h^ Clinical risk factors imply that the relevant diagnosis code(s) appeared as one of the up to 20 diagnosis codes (one principal and up to 19 additional) recorded in HIPE, a woman may have more than one clinical risk factor. The presence of a particular risk factor does not imply that this was the ‘cause’ of the CS

Source: HIPE/NPRS 2009 data

Given the large proportion of the EMCS difference accounted for by age, sensitivity analysis was carried out to test the relationship of the effect of age to the clinical risk factors in our models. Two additional models were run, one excluding clinical characteristics and the second excluding age (available from the authors on request). Excluding clinical characteristics increased the proportion explained by the model to 87.3 % with the proportion explained by age also increasing. The proportion explained increases because the prevalence of each risk factor is higher for women in the public model of care. By excluding age from the model the proportion of the difference explained by the model almost halves to 44.7 %. The proportion explained by the remaining characteristics does not change greatly. These analyses suggest that the effect of age is independent of the effect of the clinical risk factors and that the latter does not explain the age effect.

## Discussion

In Ireland and internationally studies have found that women treated privately have a higher risk of both ELCS and EMCS compared to women treated publicly [19, 20, 39]. The purpose of this paper was to ascertain if these differences could be attributed to the differing distribution of maternal, baby and hospital characteristics between the two groups.

Using national level data for Ireland these analyses showed, that a little over 50 % of the difference for ELCS could be accounted for by differences in the characteristics of public and private women. Almost 80 % of the difference in the EMCS rates across the models of care could be accounted for. The majority of the difference for EMCS is accounted for by differences in average maternal age between women experiencing public and private models of care.

The difference in the prevalence of ELCS between women experiencing public and private models of care thus largely reflects the decisions of some women to choose the private model of care and the subsequent impact age has on clinical choices by the obstetrician and the care pathway that the woman follows. Indeed, women may choose private care precisely because they perceive that they will be more able to make choices around their pregnancy and delivery in the private model.

**Fig. 2:**
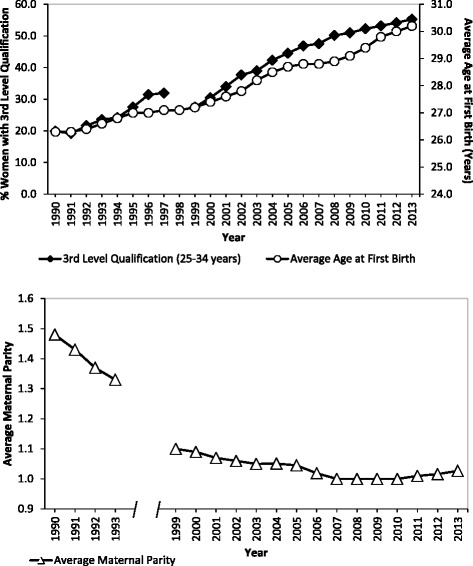
Average Age at First Birth, Percentage of Women with a 3rd Level Qualification, and Average Maternal Parity in Ireland. Note: No published NPRS data are available for 1994–1998 inclusive. Sources: Education: CSO Women and Men in Ireland, Table 5.4 2004 and Table 4.4 2013. Consistent series not available prior to 1999. Age: CSO Statbank, Vital Statistics VSA17. Parity: NPRS Annual Reports, 1990–2013

Women using the private model of care tend to be older on average because of their higher levels of education and occupational attainment (the latter shown in Table 1). Over the past decades average age at first birth in Ireland has increased, from 26.3 years in 1990 to 30.2 years in 2013, largely as a result of the higher proportion of women participating in third level education (Fig. 2). Women are staying in education for longer and are older when they are established in the labour market and in a position where they then choose to have children. Older age at first birth has led to a fall in maternal parity (Fig. 2). Increased complications associated with older maternal age are counter-balanced to some extent by the advantaged socio-economic profile of this group but nonetheless, older age at birth contributes in large part to the higher prevalence of both ELCS and EMCS among women using the private model.

There may also be other factors which could explain the difference in prevalence of CS between women using public and private models for which we do not have measures in our data set. These include maternal pre-pregnancy weight, pregnancy weight gain, fertility treatment, and staff present at delivery [12, 40–43]. However, their inclusion in the models would only increase the proportion explained if these factors were more prevalent in private patients and associated with a higher probability of CS. High pre-pregnancy weight is not actually more prevalent in private patients and although fertility treatment is more prevalent in private patients the proportions are low [20]. A second possible explanation is that there may be behavioural differences on the part of private women and/or their obstetrician that could account for these differences, although this does assume that these behaviours are not correlated with any of the characteristics analysed here.

A limitation of the data is the current lack of availability of data post-2009 that contains all of the variables required to repeat the analysis for a more recent time period. There has been a number of developments post-2009 that may have had an impact on the difference in CS rates across models of care.

Firstly, between 2009 and 2013, the downturn in the economy was reflected in a fall from 28 % to 19 % in the proportion of patients who had private status during their delivery episode. The downturn impacted more acutely on delivery discharges than non-delivery in-patient discharges where the fall in the proportion of private discharges over the period was just 3.3 percentage points. The difference here likely reflects the fact that the majority of PHI policies do not cover the entire consultant fee for maternity cases and during times of austerity even those who continued to pay their PHI premium could find it difficult to pay the additional consultant fee for private maternity care which can be over €3,000.

Over the same 5-year period, the ELCS rates for public and private patients have continued to increase (+40.9Δ%, +38Δ% respectively). The latest available national data for 2013 reports an ELCS rate of 13.1 % for public patients and 24.4 % for private patients. This marks a continued increase in the differential between the two groups [44–47]. Provisional work carried out on 2013 data with a more limited set of variables (excluding social class, nationality, birthweight, and previous miscarriage) shows little change in the proportion of the difference for ELCS explained (53.3 %) and an increase of 9 percentage points in the proportion explained for EMCS. Sensitivity analyses using this limited set of variables for 2009 found no difference to the proportion of the difference explained (53.1 % in the elective model and 78.7 % in the emergency model) giving us confidence that the more complete analyses using data from 2009 are robust.

Secondly, in June 2010, the Health Service Executive which funds maternity hospitals introduced Clinical Care Programmes for all specialities, including obstetrics and gynaecology. The purpose of the Programmes was to improve clinical input into the health services based on the key metrics of quality, financial value, access and compliance. As part of the Programme, national clinical guidelines have been rolled out including ones on obesity and pregnancy, delivery after caesarean section, and management of pre-eclampsia and eclampsia [48–50]. In addition, each maternity unit has received their own perinatal outcomes (from the NPRS) and performance tables have been developed for CS rates [51]. It is also planned to develop hospital standardised CS rates to allow maternity units to benchmark themselves nationally. It remains to be seen, however, if these quality improvement innovations lead to the standardisation of care and to the reduction in variations in CS rates across models of care.

It is difficult to predict how these developments have impacted on the model of care differential in the ELCS and EMCS rate. It is hoped that future work as part of the same project using more recent data, will go some way to explaining this.

## Conclusions

Previous literature has described the increasing prevalence of CS and the role of the private model of care in this. The purpose of this study was to quantify the differential in ELCS and EMCS rates across models of care for singleton nulliparous women and to shed light upon why these differences exist. This is interesting because Ireland is unusual in that private and public patients are delivered in the same delivery suites with the same midwifery staff working with common clinical practices and guidelines. Using national level data from all public hospitals in Ireland, covering almost 97 % of singleton nulliparous births, we find that the majority of the difference in EMCS (80 %) rates across models of care can be explained by differing characteristics between the two groups of women particularly age. However, the explained component of the difference for ELCS (53 %) is smaller; an excess private effect remains unexplained after accounting for maternal, clinical, and hospital characteristics. Additional, possibly qualitative, studies are recommended to further investigate the reasons for this large and increasing differential.

## Abbreviations

ACHI, *Australian Classification of Health Interventions*; ACS, Australian Coding Standards; CS, caesarean section; ELCS, elective caesarean section; EMCS, emergency caesarean section; HIPE, Hospital In-Patient Enquiry Scheme; ICD-10-AM, *the international statistical classification of diseases and related health problems*, tenth revision, Australian modification; NPRS, National Perinatal Reporting System

## Additional file

Additional file 1: Clinical Codes for Risk Factor Identification. ICD-10-AM/ACHI codes for identification of clinical risk factors in the data. (DOCX 13 kb)
