# Supplementary material for: Differences in nulliparous caesarean section rates across models of care: a decomposition analysis
Source: BMC Health Serv Res. 2016 Jul 8;16:239. doi: 10.1186/s12913-016-1494-3 (PMC4938942; doi:10.1186/s12913-016-1494-3)
Supplement: Additional file 1: — Clinical Codes for Risk Factor Identification. ICD-10-AM/ACHI codes for identification of clinical risk factors in the data. (DOCX 13 kb) [file 12913_2016_1494_MOESM1_ESM.docx]

| **Clinical Risk Factors** | **ICD-10-AM/ACHI Block** |
| --- | --- |
| Breech | O32.1, O64.1 |
| Diabetes (pre-existing) | E10, E11, E13, E14, O24.0, O24.1,O24.2, O24.3 |
| Eclampsia or pre-eclampsia | O14, O15 |
| Gestational diabetes | O24.4, O24.9 |
| Hypertensive disorders | O10, O11, O13, O16, I10 |
| Malpresentation | O32 (excl. O32.1, O32.5), O33 |
| Placenta praevia or placental abruption | O44, O45 |
| Poor fetal growth | O36.5 |
| Induction | Block 1334 |
